# "Apples and Oranges": Examining Different Social Groups’ Compliance With Government Health Instructions During the COVID-19 Pandemic

**DOI:** 10.34172/ijhpm.2021.21

**Published:** 2021-04-01

**Authors:** Talia Goren, Dana R. Vashdi, Itai Beeri

**Affiliations:** Division of Public Administration and Policy, School of Political Sciences, University of Haifa, Haifa, Israel.

**Keywords:** COVID-19, Minorities’ Compliance, New Health Instructions, Trust in Government, Compliance-Enhancing Factors, Israel

## Abstract

**Background:** The coronavirus outbreak has demonstrated the crucial effect of the public’s compliance with the government’s health instructions on the population’s health. However, evidence shows that some communities are less likely to comply with such instructions than others. This study highlights the factors related to intentions to comply with newly issued health directives during an ongoing extreme crisis, such as the current pandemic. In addition, it compares the impact of these factors on different minority groups and the general population in Israel.

**Methods:** Using an online survey (N=1005), we examined the impact of compliance-related factors on compliance intentions with newly issued health directives in two minority groups in Israel: the ultra-Orthodox Jewish community (N=323) and the Arab community (N=361), as well as in the general population (N=321), during the first outbreak of coronavirus disease 2019 (COVID-19). Participants were presented with a new made-up COVID-19-related instruction simulated to be issued by the Israeli Ministry of Health. Compliance intentions and compliance-related factors were measured.

**Results:** The Arab minority expressed greater intentions of complying with the instructions than the other groups. Perceptions on risk and the effectiveness of the instruction were the only two significantly associated factors with compliance intentions in all of the social groups. Additional factors affected different groups to different extents. Trust in government was related to compliance intentions only in the Arab minority.

**Conclusion:** Intentions to comply with health instructions during a crisis differ in various minority groups and in comparison to the general population, both in their levels and in the factors related to them. Policy-makers and health authorities should consider providing information about the risks and negative outcomes of the crisis as well as the expected effectiveness of the recommended behaviors. Future research should examine other minority groups and other types of instructions in different stages of a crisis.

## Background

**Key Messages**

** Implications for policy makers**

- Minority groups vary in their intentions to comply with government health instructions compared to the general population. While some groups may be more compliant, others may be equally or less so.
- Risk perceptions and the perceived effectiveness of the instruction are two variables associated with compliance in all social groups. When group-specific information is unavailable, these elements should be emphasized in public statements promoting the desired health behavior.
- Trust in government may not be a compliance enhancer in all social groups. Hence, the compliance of groups with more trust in government should not be taken for granted and may call for other compliance-enhancing strategies.

** Implications for the public**

The study will benefit the public because policy-makers will know which elements to make more salient when trying to increase the compliance of different social groups. In addition, for all social groups, policy-makers should provide more information regarding the risks posed by the crisis and emphasize why specific instructions are likely to have better consequences for the public.

The global coronavirus outbreak has demonstrated the crucial role of the public’s compliance with health instructions issued by the government for the health and safety of the population. Nonetheless, citizens do not always comply with the government’s health instructions, despite constant and repeated statements to do so through multiple communication channels and by various public figures.^1^ In particular, the compliance patterns of minority groups have attracted the attention of researchers,^2,3^ health authority representatives^4^ and the media,^5,6^ because it seems that specific communities are less likely to comply than others.^7,8^ These differences are due to cultural and socio-economic factors, such as cultural norms and practices, low income, remote living areas and poor accessibility to health information and services.^8^

 Previous studies present inconsistent evidence regarding minorities’ compliance with heath instructions. While some indicate that such groups are less likely to comply with public health directives,^9,10^ others demonstrate the opposite.^11^ The current pandemic reveals a similar dichotomy. For example, the ultra-Orthodox Jewish community in Israel and in the United States has reportedly violated coronavirus disease 2019 (COVID-19) related health instructions, such as social distancing, to a greater extent than the general population.^3,5^ Yet, other minority groups seem to behave differently. For example, American Blacks have maintained social distancing more than American Whites.^12,13^ This variance in minority groups’ compliance with health instructions highlights the importance of not assuming that all minorities will behave in the same manner.

 The theory of the cultural construction of clinical reality^14^ posits that affiliation with a cultural and/or ethnic minority group is a factor that affects people’s perceptions about health and their resulting behaviors. Consequently, different cultural minorities are likely to vary in their *extent of compliance* with health directives. In addition, various and different factors, may affect their level of compliance, based on culturally driven perceptions. The evident variance in minorities’ behavior during the COVID-19 outbreak underscores the need to understand the factors related to the compliance of different minority groups, particularly during extreme, prolonged and unprecedented global crises such as the current pandemic.^15^ Furthermore, understanding these differences is particularly important given the frequently changing health directives issued by governments and health authorities during the current pandemic,^16^ making compliance an accumulative and ongoing matter. Thus, it is important to understand not only what affects compliance with a given directive but also what may affect the willingness to comply with newly issued health directives amidst an ongoing crisis, which involves previous and frequent issuing of directives. To the best of our knowledge, previous studies have not identified the factors that promote or impede compliance intentions with newly issued instructions, particularly among minority groups. By doing so, this study will allow policy-makers and health authorities to adjust their crisis-controlling policies and communication strategies to different social groups and enhance public compliance.

 In this study, we examined the relationship between several key compliance-related factors and intentions to comply with a simulated newly issued heath directive in two distinct minority groups in Israel, the ultra-Orthodox and Arab communities, as well as in the general population, during the first outbreak of COVID-19 (April 2020). The ultra-Orthodox Jewish community in Israel accounts for 11% of the Israeli population.^17^ It is a defined minority group, committed to Jewish religious law. It is composed of several sub-groups, which segregate themselves from the secular environment of the Israeli majority. Furthermore, the ultra-Orthodox Jewish community is very conservative and actively preserves ancient traditions and obedience to rabbinical authority. Consequently, a significant part of this community lives in isolated areas and neighborhoods, and operates separate educational systems.^18-21^ Furthermore, it is traditionally associated with right-wing parties and its sectorial ultra-Orthodox parties have joined the right-wing coalition in most Israeli governments in recent decades.^22^

 The Arab community accounts for 20% of Israeli society.^17^ It is a significant ethnic and religious minority that does not accept the Jewish nature of the state and identifies with residents of the Palestinian territories. Most of its members do not assimilate into the Jewish majority and reside in Arab villages, towns and cities, with a separate educational system.^23-25^ The Arab minority traditionally supports left-wing parties and its sectorial parties have never joined the coalition in the Israeli governments.^22,24,26^

 A plethora of studies have pointed out various factors associated with compliance with preventive health measures during the COVID-19 pandemic^1,27-33^ and in previous ones.^11,34-40^ Such factors include demographic factors such as gender, age and education. The studies have noted that women, older people and people with more education are usually more compliant.^1,11,27,28^ These studies have also indicated that psychological and psychosocial factors, including emotional distress, risk perceptions and perceptions about the efficacy of the instructions and one’s ability to comply with them are all positively associated with compliance.^1,11,27,29,35–37,41^ Additional factors that affect compliance are political and civic attitudes such as political orientation, trust in government and the health authorities, legal and moral norms, as well as economic conditions and considerations.^1,29,38,39,42-46^

 Moreover, studies have found complex relationships between the factors associated with compliance. For example, Roma et al^1^ focused on the Italian population during the first outbreak of the COVID-19 virus, and found that the association between the perceived efficacy of the governmental health directives and compliance depended on people’s risk perceptions. Shao and Hao^46^ reported that American liberals felt the virus posed more of a risk than conservatives did, and that confidence in political figures explained the relationship between political views and risk perceptions.

 The above-mentioned factors are often framed within the health belief model,^47^ which is the most commonly used theoretical framework for predicting health behavior.^48^ The model emphasizes the effect that one’s beliefs and perceptions regarding health behaviors and measures (eg, their benefits and risks) have on one’s health behavior.^49,50^ A fundamental assumption of the model posits that the level of perceived risk posed by a health threat and the perceived efficacy (effectiveness) of a specific behavior in minimizing this threat are positively associated with the likelihood of a person engaging in the behavior.^47,51^ In other words, the greater the threat a person believes a disease is and the more effective that person believes a behavior is in minimizing the risk of contracting the disease, the more likely the person is to adopt the suggested behavior. These considerations are rudimentary for predicting one’s health behavior, as they tap into the basic rationale of the human cognitive process. Other theoretical frameworks explaining health behaviors, such as the protection motivation theory, refer to these considerations as well.^52^ Though minority groups may regard health hazards as riskier than the majority group, due to an increased sense of vulnerability,^46,53-56^ the *nature* of the relationship between risk perceptions and compliance with suggested health measures in minority groups should be similar to the one in other social groups. Hence, we predict that perceptions regarding the risks posed by the Coronavirus will be positively associated with compliance intentions in all social groups universally. Similarly, the perceived effectiveness of the instructed health behavior is likely to be related to compliance intentions in the same manner in all social groups.

 However, while some compliance-related factors may affect all social groups, majority and minority alike, in the same manner, other factors may affect minority groups differently. One factor that may be relevant for predicting compliance with health guidelines among minority groups is the level of trust in government. Trust in government is reported to be a key factor in promoting compliance with health instructions.^34,38,39,42,57^ Minority groups often trust the government less than the majority group. Several explanations for this lack of trust include their lack of integration and representation in government institutions and their limited political efficacy.^3,58-60^ As a result, minorities may be less likely to comply with official health instructions than groups with more trust in the government.

 Nevertheless, these overall low levels of trust in government in minority groups do not necessarily mean that the relationship between trust in government and compliance in these groups will be weaker than in the general population. On the contrary, for social minorities, particularly ethnic and national minorities that do not feel a sense of belonging to the majority population, the correlation between trust in government and compliance may be stronger than in the general population because of their national identification.^61^ While the majority group shares the same national identity as the government, and identifies with it as representing its nationality (at least to some extent), ethnic and national minority groups identify less with the majority government and often feel alienated from it and by it.^61-63^ This sense of identification vs. alienation may affect the role that trust in government plays in compliance with the government’s directives. When identification with the majority government is high, trust may have a weak association with compliance because such identification with national institutions overshadows trust in the specific government ruling at the moment. As a result, those who identify with the national government in general are willing to comply with directives from it regardless of the level of trust they have in the current administration. However, when identification with the national institutions and the majority government is low, trust may have a strong association with compliance. Put differently, if those who do not identify with the government trust it, they are more likely to comply with its directives than people who trust the government less or identify with it more.

 In Israel, both ultra-Orthodox Jews and Arab groups do not identify with the government and its institutions.^25,64,65^ In the case of the ultra-Orthodox community, scholars have attributed these sentiments to the community members’ lack of willingness to integrate with the secular majority and their commitment to Jewish law over state law.^65-67^ The low levels of identification with the government in the Arab community are attributed to the feeling that the Israeli government discriminates against their community members due to their ethnic background and their support of the Palestinian side in the Israeli-Palestinian conflict.^64,68^ Furthermore, as mentioned above, historically and unlike ultra-Orthodox parties, Arab parties have not been part of the ruling coalition of the national government.^26^ This lack of representation makes it especially hard for them to identify with the government (in accordance with the “political empowerment” paradigm,^58,69-71^). Hence, given the deep lack of identification of the Arab minority^64^ and according to the logic provided above, we hypothesized that the intentions of members of the Arab minority to comply with health directives would be more strongly related to their trust in the government in comparison to the Jewish groups.

 Another compliance related factor that we suggest might affect compliance intentions in minority groups differently than in the majority group is the ability to comply with the instructed behavior. More specifically, we posit that for members of social groups that regard the government’s policies as inappropriate for their lifestyles and cultural practices, there will be a weaker association between their technical ability to comply with the government’s instructions and their actual intentions of doing so. Simply put, when people are used to receiving instructions that conflict with their normal way of life and their ability to comply with them, they may regard this ability as less important in their decision to comply, as they realize they are obligated to comply with these instructions regardless of their ability to do so.

 One of the most prominent examples is the demand to maintain social distancing from a family member who is in home quarantine. The Arab and ultra-Orthodox communities typically have large families and a lower socioeconomic status than the general population.^72^ Families in such communities often have many children and live in much smaller residential units, making it almost impossible to observe the home-quarantine instructions. Another example of a cultural mismatch between COVID-19 policies and the lifestyle of these minority groups is the shutdown of schools and the use of online remote learning. Both of these minority groups have limited access to the Internet and computers, which they generally cannot afford. In addition, the ultra-Orthodox community generally opposes the use of the Internet, resulting in a significant digital divide by choice.^73^ Furthermore, this community regards the demand to shut down its schools as an attack on what it seen as a major component of its lifestyle—religious study.^74^ Therefore, in practice, neither of these groups could adopt the remote schooling policies.^75,76^ These examples demonstrate that, at least initially, the government instituted policies that these minority communities were unable to implement. However, over time, members of these groups as well as the government found ways to deal with these issues to some extent. Thus, we hypothesized that the positive relationship between the ability to comply and the intention to comply would be weaker in social groups who were given previous preventive instructions that were not adjusted to their lifestyle and initially seemed impossible to comply with. In other words we predict that in the ultra-Orthodox and Arab minorities the ability to comply with instructions will have a weaker association with the intention to comply compared to the general population. Based on the existing compliance-related literature and on empirical evidence regarding minorities’ behavior in past health crises, we focused our investigation on leading social, psychological, economic and medical variables. We propose that not only will the compliance of minority groups depend on different factors than the general population, but also that at least some of these factors will vary between the minority groups. More specifically, relying on the rationales presented above, we hypothesized that trust in government and the ability to comply will impact intentions to comply in a different manner depending on the social group the individual belongs to. While previous studies have examined various factors associated with compliance in different countries as well as in Israel,^27-29,43^ they did not consider minority groups variance. Given that many countries are ethnically and culturally diverse,^8^ investigating such variations is crucial for helping improve the compliance with health directives during the current pandemic and in other health crises.

## Methods

 We conducted an online survey in which participants were presented with what appeared to be a newly issued health directive and asked to report their intentions of complying with it. We focused on compliance intentions and not on self-reported actual behavior for two reasons. First, we wanted to examine compliance with new health directives amidst an on-going crisis, in which other health instructions were issued previously. We did so because in a prolonged crisis, such as the COVID-19 pandemic, new directives are issued as knowledge about the virus and its contagion accumulates.^16^ Thus, it is important to identify the factors that are associated with compliance with new directives, above and beyond those that have already been issued. Second, we wanted to minimize the likelihood of the social desirability effect that occurs in self-reporting about normative or illegal behavior,^77-81^ and non-compliance with government health instructions could be considered as such. Social desirability is “the tendency of respondents to distort self-reports in a favorable direction.”^82^ Though both self-reported behavior intentions and self-reported behavior are associated with social desirability,^83^ by measuring compliance intentions and not actual behavior, which may have legal implications, we tried to reduce the effect of social desirability by eliminating the possibility of self-incrimination. Furthermore, behavioral theory research, and particularly studies that focus on Ajzen’s theory of planned behavior,^84^ suggests that intentions and behavior are directly associated.^85^ Thus, compliance intentions are often measured in studies trying to predict and analyze behavior and actual compliance with preventive health measures,^35,36,38,86-89^ including in the current pandemic.^28,29,90^ Hence we chose to apply this method as well.

###  Sample

 One thousand and five participants took part in the survey, 321 from the general population, 323 from the ultra-Orthodox Jewish community and 361 from the Arab community. Participants were recruited through two local panel survey companies in order to obtain a sufficient sample of the Arab minority, given its low response rates.^91^ We used data from the Central Bureau of Statistics in Israel to stratify the general population and Arab minority samples by age and gender. Given that many people in the ultra-Orthodox community reject the use of the Internet for religious reasons,^18^ we stratified the ultra-Orthodox sample by gender alone. We adopted this approach despite the fact that doing so probably resulted in an underrepresentation of the older ultra-Orthodox community. Descriptive statistics of the demographic distribution of the sampled populations are presented in Table 1.

**Table 1:** The Means and Standard Deviations of the Demographics by Social Group

**Variable **	**Arab Minority (N = 361** ^a^ **)**		**Ultra-Orthodox (N = 323** ^b^ **)**		**General Population (N = 321** ^c^ **)**	
	**Mean**	**Standard Deviation**	**Mean**	**Standard Deviation**	**Mean**	**Standard Deviation**
Gender^d^	1.61	0.489	1.52	0.500	1.43	0.496
Age	35.24	10.974	28.40	7.157	43.56	16.619
Number of children	1.50	1.446	2.17	2.418	1.78	1.724
Income level^e^	1.86	1.122	1.89	1.043	2.46	1.221
Education^f^	1.67	0.472	1.50	0.501	1.59	0.493

^a^ Number of valid responses for income level was N = 344 and for education N = 353. ^b^Number of valid responses for income level was N = 283. ^c^Number of valid responses for income level was N = 283. ^d^Gender: 1 = male, 2 = female. ^e^Income level:relative to average household income: 1 = “much lower” to 5 = “much higher,” 0 = “no income.” ^f^Education: 1 = high school education and below, 2 = above a high school education.

 At the onset of the survey (April 23, 2020), the daily increase in infected patients in Israel was 2.1%, and severe morbidity and mortality rates were 2.76% and 1.28%, respectively^[1]^. In addition, emergency regulations for social distancing including the closing of schools, workplace shutdowns and the banning of gatherings were imposed. These regulations were toughened on a daily basis.

###  Procedure

 Participants were presented with a made-up, yet plausible, COVID-19-related instruction, supposedly issued by the Israeli Ministry of Health on the morning of the survey, calling on citizens to measure their temperature once a day and report the findings to the Ministry of Health if it exceeded 38°C (100.4°F). During the period of the data collection, body temperature monitoring was mandatory in Israel prior to entering public venues such as grocery stores and healthcare clinics. In addition, citizens were asked to report any changes in their health status to their primary healthcare provider. Our made-up instruction combined these two elements, making it a reasonable order from the Ministry of Health, particularly in times of uncertainty and frequently changing new instructions. Next, participants were asked to state their intentions of complying with this directive. We then asked the participants questions regarding the factors assumed to affect their compliance intentions, as well as demographic questions. At the end of the survey the participants were informed that the instruction was bogus.

###  Measures 

####  Dependent Variable

*Intentions to comply. *We measured this variable using the protocol for measuring behavioral intentions in a medical context.^92^ The scale included three items and asked the participants to indicate the degree to which they agreed with them on a scale ranging from 1 (strongly disagree) to 5 (strongly agree). The three items are identical except for the verb regarding intentions: “I expect/want/intend to check my temperature every day and report it to the Ministry of Health if it exceeds 38°C.” The scores of the three items were averaged to a single scale, following an inter-item reliability check (Cronbach’s alpha = 0.941).

####  Independent variables

*Stress*. We used the 14 items from the perceived stress scale^93^ to assess this variable. The participants were asked to indicate the degree to which they agreed with these items on a 5-point scale ranging from 1-never to 5-very often. A sample question is: “In the last month, how often have you felt nervous and stressed?” Following an inter-item reliability check (Cronbach’s alpha = 0.887), the scores were summed and ranged from 14 (low stress) to 70 (high stress).

*Ability to comply*. A single yes/no (1/0) question asked the participants whether they had a working thermometer at home.

*Perceived effectiveness (efficacy)* of the instruction. We asked the participants: “To what extent do you assume that complying with the directive of the Ministry of Health will help the fight against the coronavirus?” Responses ranged from 1 = “very little extent” to 5 = “very great extent.”

*Social capital*. We used the Martin et al scale for social capital^94^ to assess this variable with seven items. We asked the participants to indicate their agreement with the items on a 4-point scale: (1) strongly agree; (2) agree; (3) disagree; (4) strongly disagree. Following the original scale, for each item, answers 1+2 were coded as 1 and answers 3+4 were coded as 0. The total score was 0-7. A sample item is: “People in this neighborhood can be trusted.”

*Trust in government. *We used the 5-item cynicism/trust scale to measure the level of trust in assessments about the professional and personal integrity of the government.^95,96^ Answers could range from 0 to 100, with 0 indicating low levels of trust. Following an inter-item reliability check (Cronbach’s alpha = 0.891), items were averaged to form a single scale ranging from 0 to 100. A sample item is: “How much of the time do you think you can trust the government to do the right thing?”

*Knowledge about the disease*. We used six questions inspired by the 2009 H1N1 influenza knowledge measure.^97^ The questions focused on familiarity with symptoms, modes of transmission, incubation period and preventive measures. Correct answers were summed to a maximum of 20 points.

*Satisfaction with the government’s performance during the pandemic*. We asked the participants: “To what extent are you satisfied with the government’s performance regarding the coronavirus crisis in the last month?” They responded on a 5-point scale item ranging from (1) very little extent to (5) very great extent.

*Medical risk perceptions about the virus*.^35,98,99^ We used three items to measure both cognitive and emotional perceptions about the risk: “How likely do you think it is that you would become sick with coronavirus?” (1 = not at all likely – 5 = very likely); “How severe would your condition be if you did become sick with coronavirus?” 1 = not at all – 5 = very severe); and “To what extent are you worried about the possibility of becoming sick with coronavirus?” (1 = not at all to 5 = very much). Following an inter-item reliability check, which, despite its relatively low value is still considered acceptable^100,101^ (Cronbach’s alpha = 0.654), the scores were averaged to form a single scale ranging from 1 to 5.

*Financial risks posed by the pandemic*. We used the same measure for assessing perceptions about health risks, adjusted to reflect financial risks (Cronbach’s alpha = 0.879).

####  Control Variables

 We controlled for gender, age and number of children.We also controlled for income by asking the participants to indicate ona 5-point scale whether their own household income level was (1) much lower to (5) much higher than the average household income or they had no income at all (0). Finally, we also asked the participants if they had an education beyond the high school level (1 = no; 2 = yes)

###  Statistical Analysis 

 We conducted an analysis of variance to examine the difference in the mean levels of compliance intentions among the three social groups: the Arabs, the ultra-Orthodox and the general population. The relationship between the independent variables and compliance intentions was tested using an ordinary least square (OLS) hierarchical regression analysis in each of the social groups. In these regressions the variance inflation factor values were lower than 5 and the tolerance indexes were neither close to 0 nor above 1, which ruled out any concerns about multicollinearity.^102^ To test whether the relationship between specific independent variables and compliance intentions were contingent on social group, we added interactions between these variables and the social group in an OLS regression that included the entire sample.

## Results

Table 2 presents the means of the measured variables in each of the groups^[2]^. The mean compliance intentions score of the overall sample was 2.78 on a scale of 1 to 5 (SD = 1.33). Statistically significant differences in compliance between the examined social groups emerged. On average, the Arab participants demonstrated the highest compliance intentions (3.17). The mean level of compliance intentions in the ultra-Orthodox minority was similar to that of the general population (2.6 and 2.53, respectively; *F*_(2,1002)_ = 25.140; *P*< .001). To examine the relationship between social group affiliation and compliance intentions further, we conducted a hierarchical regression with two models. The first included the control variables (ie, gender, children, income, education, and age), and the second included two dummy variables indicative of the three social groups. The second model explained 5% more of the variance in compliance, demonstrating the mild, but significant effect of social group affiliation on compliance intentions.

**Table 2:** The Means and Standard Deviations of the Measures by Social Group

**Variable **	**Arab Minority (N = 361)**		**Ultra-Orthodox (N = 323)**		**General Population (N = 321)**	
	**Mean**	**Standard Deviation**	**Mean**	**Standard Deviation**	**Mean**	**Standard Deviation**
Stress	40.41	9.674	39.77	9.604	38.34	9.214
Compliance ability	0.85	0.355	0.90	0.299	0.93	0.253
Perceptions about effectiveness	3.16	1.262	2.86	1.105	2.80	1.184
Social capital	5.17	1.727	6.01	1.460	4.93	1.829
Trust in government	34.35	23.242	51.33	24.149	37.84	25.924
Knowledge	15.98	4.162	17.44	2.135	17.33	2.538
Satisfaction	2.60	1.093	3.72	0.960	3.09	1.211
Medical risk perceptions	2.82	0.868	2.30	0.719	2.66	0.869
Financial risk perceptions	3.68	1.033	3.33	1.079	3.58	1.118
Compliance	3.17	1.333	2.60	1.299	2.53	1.265

 Comparing the mean levels of trust in government and risk perceptions revealed interesting results. As expected, on average the Arab group expressed the least trust in the government (M = 34.35), but unexpectedly the ultra-Orthodox group expressed the most trust (M = 51.33; *F*_(2,1002)_ = 44.958; *P*< .001). With regard to risk perceptions, on average the ultra-Orthodox group had the lowest level (M = 2.3 out of 5), while the Arab group had the highest level (M = 2.82; *F*_(2,1002)_ = 34.453; *P*< .001).

 Another review of the descriptive data in Table 2 indicates that one explanation for the Arab minority’s high levels of compliance intentions might be the high levels of compliance-associated variables this group reported. For example, they had higher levels of stress and stronger perceptions regarding medical and financial risks. However, this group also expressed little trust in the government and lower levels of knowledge about the disease, variables that have in the past been shown to be positively associated with compliance. Hence, in order to assess the actual effect of these variables on compliance intentions, we performed an in-depth analysis of the association between these variables and compliance intentions in each of the sectors.

###  Compliance-Related Factors in Different Social Groups

 We conducted an OLS regression analysis for each of the social groups (the two minorities and the general population), while controlling for the demographic variables.

####  The General Population

 As Table 3 indicates, perceptions regarding the effectiveness of the regulation and risk perceptions were significant and positively related to this group’s compliance intentions (b = 0.594; *P*< .001; b = 0.182; *P*< .01, respectively; with standardized beta coefficients of β = 0.556 and β = 0.125). The more the participants regarded the directive as effective in dealing with the pandemic and the riskier the virus was perceived to be, the stronger the intentions to comply were. The Pearson correlations of these variables and compliance were also positive and significant (r(effectiveness) = 0.567; *P*< .001; r(risk perceptions) = 0.151; *P*< .01, respectively). One factor that was related to compliance intentions only in this group was the ability to comply with the regulation (b = 0.605; *P*< .05; with standardized beta coefficients of β = 0.127). In other words, people who did not feel that they had the ability to comply with the directive, meaning they did not have a working thermometer at home, were more likely to have weaker intentions of doing so. The Pearson correlations of this variable and compliance intentions was also positive and significant (r = 0.127; *P*< .05). As Table 3 shows, the model including all the above-mentioned variables explained 35.2% of the variance in compliance intentions above and beyond the model with only the control variables.

**Table 3:** OLS Regression Analysis by Social Group

	**Arab Minority (N = 361)**				**Ultra-Orthodox (N = 323)**				**General Population (N = 321)**			
**Dependent variable: Compliance**												
	**Model 1**		**Model 2**		**Model 1**		**Model 2**		**Model 1**		**Model 2**	
	**B**	**SE**	**B**	**SE**	**B**	**SE**	**B**	**SE**	**B**	**SE**	**B**	**SE**
(Constant)	4.219^c^	0.455	0.624	0.594	2.923^c^	0.590	0.233	0.900	3.147^c^	0.390	1.178	0.690
Gender	-0.305^a^	0.149	-0.128	0.111	-0.011	0.180	0.027	0.160	-0.204	0.155	-0.042	0.129
Kids	0.013	0.053	-0.021	0.040	-0.008	0.054	0.057	0.048	-0.106^a^	0.053	-0.074	0.046
Income	-0.130	0.067	-0.121^a^	0.051	0.116	0.079	0.124	0.070	-0.017	0.066	-0.026	0.056
Education	-0.436^b^	0.157	-0.298^a^	0.120	-0.007	0.173	-0.070	0.149	-0.160	0.173	-0.144	0.145
Age	0.011	0.007	0.010	0.005	-0.017	0.019	-0.019	0.016	0.004	0.006	0.001	0.005
Stress			-0.004	0.006			-0.004	0.008			-0.010	0.008
Compliance ability			0.286	0.154			-0.284	0.233			0.605^a^	0.239
Perceived effectiveness			0.601^c^	0.045			0.624^c^	0.062			0.594^c^	0.055
Social capital			0.053	0.033			0.045	0.047			-0.033	0.035
Trust in government			0.010^b^	0.003			-0.004	0.003			0.003	0.004
Knowledge			0.009	0.014			0.038	0.034			0.005	0.026
Satisfaction			-0.049	0.057			0.014	0.087			-0.119	0.077
Medical risk perception			0.277^c^	0.070			0.219^a^	0.098			0.182^a^	0.077
Financial risk perception			-0.062	0.057			0.004	0.069			-0.063	0.062
R^2^	0.064		0.506		0.013		0.322		0.030		0.382	
ΔR^2^	0.064^b^		0.442^c^		0.013		0.309^c^		0.030		0.352^c^	

Abbreviations: OLS, ordinary least square; SE, standard error. ^a^ *P *< .05, ^b^*P *<.01, ^c^*P *<.001.

####  The Arab Minority 

 As Table 3 indicates, in the Arab group, three factors, aside from demographic variables, were significantly and positively associated with compliance intentions: perceptions about the effectiveness of the regulation, trust in government and risk perceptions (b = 0.601; *P*< .001; b = 0.010; *P*< .01; b = 0.277; *P*< .001, respectively, with standardized beta coefficients of β = 0.569, β = 0.170 and β = 0.177, respectively). The more the participants regarded the directive as effective in dealing with the pandemic, the more they trusted the government, and the riskier the virus was perceived to be, the stronger their intentions to comply were. The Pearson correlations of these variables and compliance were also positive and significant (r(effectiveness) = 0.639; r(trust) = 0.324; r(risk perceptions) = 0.198; *P*< .001). As Table 3 illustrates, the model including all of these variables explained 44.2% of the variance in compliance intentions above and beyond the model with only the control variables.

####  The Ultra-Orthodox Minority

 In this group only perceptions regarding the effectiveness of the regulation and risk perceptions were significantly and positively associated with compliance (b = 0.624; *P*< .001; b = 0.219; *P*< .05, respectively, with standardized beta coefficients of β = 0.533 and β = 0.122, respectively). In other words, the more the ultra-Orthodox participants regarded the directive as effective in dealing with the pandemic, and the riskier the virus was perceived to be, the stronger their intentions to comply were. The Pearson correlations of these variables and compliance were also positive and significant (r(effectiveness) = 0.532; *P*< .001; r(risk perceptions) = 0.161; *P*< .01). As Table 3 indicates, the model including all of these variables explained 30.9% of the variance in compliance intentions above and beyond the model with only the control variables. Note that, in this group, the ability to comply with the directive seemed to be negatively related to intentions to do so, although the relationship was not significant. When examining the Pearson correlation between these two variables, we found a very weak negative relationship that was also non-significant (Pearson’s r = -0.04).

 Next, in order to examine whether the differences between the sectors in the variables related to compliance intentions were statistically significant, we conducted a final analysis in which we included all of the above-mentioned variables and their interaction with social group (using dummy variables with the reference group being the general population). First, we examined the effect of all of the control variables on compliance intentions and then added the variables that were found to be associated with compliance in each of the social groups (Table 4, models 1+2). Next, we tested for interaction effects between social group and each of the variables that were found to be associated with compliance in each of the groups (models 3-6). Finally, as a robustness test for the interaction effects that emerged, we ran a full interaction model that included all of the above-mentioned interactions to determine the impact of each interaction above and beyond all of the other interactions (model 7).

**Table 4:** The Results of the OLS Regression and Analysis of the Interactions of Variables Significantly Related to Compliance and Social Group

	**Model 1**		**Model 2**		**Model 3**		**Model 4**		**Model 5**		**Model 6**		**Model 7**	
**Dependent Variable: Compliance**														
	**B**	**SE**	**B**	**SE**	**B**	**SE**	**B**	**SE**	**B**	**SE**	**B**	**SE**	**B**	**SE**
(Constant)	3.080^c^	0.263	0.285	0.273	0.026	0.326	0.373	0.301	0.442	0.286	0.400	0.305	0.286	0.370
Gender	-0.155	0.090	-0.063	0.072	-0.064	0.072	-0.063	0.072	-0.076	0.072	-0.058	0.072	-0.074	0.072
Children	-0.041	0.027	-0.010	0.022	-0.010	0.022	-0.011	0.022	-0.009	0.022	-0.010	0.022	-0.011	0.022
Income	-0.039	0.040	-0.021	0.032	-0.023	0.032	-0.020	0.032	-0.018	0.032	-0.021	0.032	-0.022	0.032
Education	-0.232^a^	0.094	-0.198^b^	0.076	-0.201^b^	0.076	-0.196^a^	0.076	-0.179^a^	0.076	-0.197^a^	0.076	-0.180^a^	0.076
Age	0.005	0.004	0.005	0.003	0.005	0.003	0.005	0.003	0.004	0.003	0.005	0.003	0.005	0.003
Social group- UO	0.149	0.130	0.138	0.105	0.779^a^	0.303	0.074	0.244	0.275	0.191	0.028	0.290	0.739	0.457
Social group- Arab	0.676^c^	0.113	0.447^c^	0.092	0.649^a^	0.267	0.276	0.224	0.081	0.153	0.177	0.280	0.079	0.408
Compliance ability			0.222	0.113	0.524^a^	0.232	0.220	0.113	0.202	0.112	0.223^a^	0.113	0.543^a^	0.232
Perceived effectiveness			0.619^c^	0.030	0.615^c^	0.030	0.588^c^	0.053	0.613^c^	0.030	0.619^c^	0.030	0.602^c^	0.053
Trust in government			0.002	0.001	0.002	0.001	0.002	0.001	-0.001	0.002	0.002	0.001	-0.001	0.002
Medical risk perceptions			0.191^c^	0.043	0.190^c^	0.043	0.191^c^	0.043	0.187^c^	0.042	0.140	0.072	0.136	0.071
UO X Compliance ability					-0.702^a^	0.312							-0.718^a^	0.312
Arab X Compliance ability					-0.211	0.283							-0.281	0.282
UO X Effectiveness perceptions							0.023	0.077					0.012	0.078
Arab X Effectiveness perceptions							0.057	0.069					0.010	0.070
UO X Trust in government									-0.002	0.003			-0.002	0.003
Arab X Trust in government									0.010^b^	0.003			0.010^b^	0.003
UO X Medical risk perceptions											0.020	0.112	0.061	0.112
Arab X Medical risk perceptions											0.078	0.097	0.090	0.097
R^2^	0.062		0.402		0.406		0.403		0.412		0.403		0.417	

Abbreviations: OLS, ordinary least square; SE, standard error; UO, ultra-Orthodox. *Note.* N = 901; Reference group: General population. ^a^ *P *< .05, ^b^*P *<.01, ^c^*P *<.001.

 As Table 4 shows, the only significant interactions with social group were trust in government and the ability to comply with the instruction. Aside from these two variables, all of the other variables were related with compliance intentions in the same manner across all social groups. Two of these variables are perceptions about the risk posed by the disease and the effectiveness of the instructed behavior, which had a positive association with compliance intentions in all social groups. This finding supports our first hypothesis.

**Figure 1:**
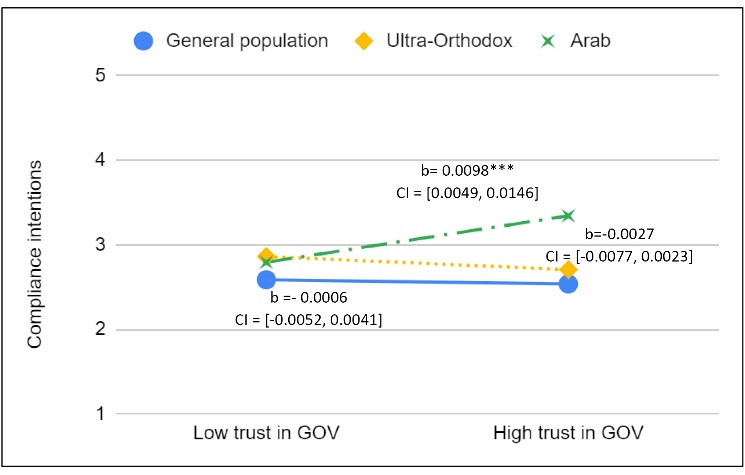
Interaction Effect of Social Group on the Relationship Between Trust in Government and Compliance Intentions.

 The interaction between trust in government and social group indicated that, in the Arab group, the relationship between trust in government and the intention to comply was significantly different than in the general population (b = 0.010; *P <* .01; standardized beta coefficients β = 0.166). As Figure 1 illustrates, while in the Arab group trust in government *was* positively and significantly related to compliance intentions, in the general population and in the ultra-Orthodox group this relationship *was not* significant. Note that models 2-7 in Table 4 were also tested without control variables and produced similar results. This finding supports our second hypothesis that trust would be associated with compliance intentions in the Arab group to a greater extent than in the other groups. However, a closer examination revealed that the significant effect of trust in government on compliance intentions within the Arab minority was small. When examining a reduced model that included only the variables that were significantly related to compliance intentions in the Arab group, the addition of one standard deviation to the mean score of trust in government increased compliance intentions by 0.227 points (from 3.156 to 3.383 in a range of 1-5). A two standard deviation change in the mean of trust increased compliance intentions by 0.445 points (from 3.156 to 3.611). Although this is not a large effect, it is still substantial and significantly different than that in the other social groups.

 Another interesting finding emerged from this interaction analysis. As Figure 1 illustrates, while members of all social groups with low levels of institutional trust had similar levels of compliance intentions, Arabs with high levels of institutional trust had higher levels of compliance intentions compared to other group members who had high levels of trust. We suggest an explanation for these somewhat unintuitive findings in the discussion section.

**Figure 2:**
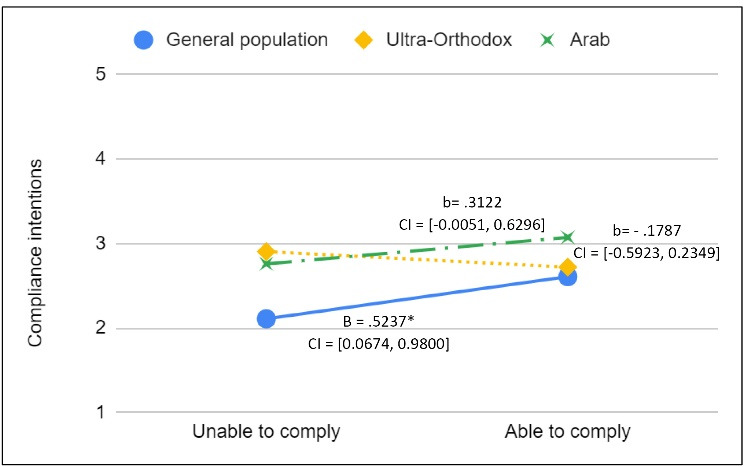
Interaction Effect of Social Group on the Relationship Between Ability to Comply and Compliance Intentions.

 The interaction between the ability to comply with the instruction and social group indicated that the relationship between the ability to comply with the instruction and the intention of actually doing so was significantly different between the general population and the ultra-Orthodox population (b = -0.718; *P*< .05; standardized beta coefficients β = -0.243). As Figure 2 illustrates, while in the ultra-Orthodox and Arab groups the ability to comply with the instruction was not related to the intentions of doing so, in the general population this relationship was significant and positive. This finding supports our third hypothesis, which posited that the ability to comply with the instruction would be associated with compliance intentions to a smaller extent in the Arab and ultra-Orthodox groups compared to the general population. However, a closer examination revealed that the significant effect of the ability to comply on compliance intentions within the general population was of a very modest size. In a reduced model that included only the variables significantly related to compliance intentions in this group, the addition of one standard deviation to the average compliance ability increased compliance intentions by only 0.123 points (from 2.532 to 2.664 in a range of 1-5). The addition of two standard deviations to the average compliance ability increased compliance intentions by 0.265 points (from 2.532 to 2.797). While this is a very subtle effect, which could be attributed to the small sample size, it is possible that this effect would increase in a larger sample size.

 Interestingly, other variables that were found to be associated with compliance with health instructions in similar situations in previous studies, such as knowledge about the disease and satisfaction with the government’s performance during the crisis, were not associated with the compliance intentions of any of the groups. In addition, education was negatively associated with compliance intentions. While this result is contrary to some previous research indicating a positive association between educational level and compliance in both previous pandemics and in the current one,^28,103^ other recent COVID-19 related studies report a null association between the two.^32,45^ This discrepancy could be caused by the specific instruction examined and the way it is perceived, as evidence shows that on the one hand education level is positively associated with preventive care activities but on the other negatively associated with health practices.^104^ More interestingly, trust in government, which is one of the main predictors of compliance with government health instructions,^39,105-107^ was not related to compliance intentions except for the Arab minority. In addition, given previous evidence regarding the mediating effect of risk perceptions on the relationship between perceptions about the effectiveness of the directive and compliance,^1^ we checked for this effect in our data. However, it was not significant in any of the examined groups.

## Discussion

 Our findings about intentions to comply with newly issued government health instructions during a pandemic not only stress the variance in minority groups’ compliance with new health instructions, but also demonstrate partial yet apparent heterogeneity in the relationships between compliance intentions and its associated factors in different social groups. While some factors are associated with compliance intentions in the same manner across all social groups, other factors demonstrate mildly different relationships with compliance intentions in different social groups.

 Specifically, our results revealed that perceptions about the risk posed by the disease and the effectiveness of the suggested measure were positively associated with compliance intentions in all social groups. However, trust in government and the ability to comply with the suggested health measure varied in their relationship with compliance intentions in different social groups. While trust was positively associated with compliance intentions only in the Arab group, it was not associated with the compliance intentions of other groups. Similarly, the ability to comply with the suggested instruction was positively associated with compliance only in the general population, not in the minority groups. These findings suggest that compliance intentions with newly instructed health behaviors during an on-going health crisis can be promoted either by group-adjusted strategies or by generic strategies that emphasize the basic aspects of health behavior motivation: perceptions of risks posed by the health hazard and the prospected effectiveness of the desired behavior. Furthermore, our findings indicate that the promotion of factors that were found to be group-specific, such as trust in government or compliance ability can be applied to all social groups, as while they are positively related to compliance in some groups, they have no effect in the other social groups (ie, there is no risk of harming compliance in the other groups).

 Our results stress the variance in minority groups’ compliance with new health instructions. In accordance with some previous research highlighting ethnic minorities’ increased likelihood of adopting health recommendations,^11^ but contrary to our expectations given the specific Arab-Israeli context, the Arab minority group had stronger intentions of complying with the regulation we presented than the general population did. In other words, despite the unique features of the Israeli case, the Arab minority in Israel demonstrated behavior in accordance with that reported regarding other minority groups in other geo-political contexts.^12,13^ Although one may attribute this finding to social desirability bias that often occurs when surveying minority groups,^108^ including the Arab minority in Israel,^109^ the mean score of the Arab group on risk perceptions, a well-established compliance-associated factor that was related to compliance intentions in our study as well, provides an alternative explanation for the high levels of compliance intentions found in the Arab group in our study. Simply put, since members of the Arab minority regarded the risks posed by the virus as greater than members of other social groups, and since this risk perception was associated with compliance intentions, they expressed greater intentions to comply with the presented preventive instruction. This increased concern about the risks posed by the virus might result from this group’s poorer access to healthcare services, partly due to the fact that they tend to live in outlying areas.^110,111^

 Indeed, the results show that minority groups regard the risks of the virus differently than the general public. They also have different levels of trust in the government that issued the preventive instructions, compared to the general population. In accordance with previous research, the Arab minority’s risk perceptions were the highest,^46,53-55^ and the ultra-Orthodox group’s the lowest,^112-114^ which can also explain the difference in their intentions to comply with the presented instruction. However, our results regarding the minority groups’ levels of trust in government do not fully coincide with the existing literature. On average, the Arab group had the least trust in the government and the ultra-Orthodox group had the most trust. The historical and political context of the Jewish-Arab relationship can explain the Arabs’ lack of trust.^26,68^ One explanation for the unusually high levels of trust in the government of the ultra-Orthodox might be that at the time the survey took place, Israel’s Health Minister, who was at the forefront of the battle against the virus, was a member of the ultra-Orthodox community. This fact may also explain the high levels of satisfaction this community expressed with the government’s performance during the pandemic.

 We hypothesized that the positive relationship between risk perceptions and perceptions about the effectiveness of the health directive would not vary across social groups, given the basic and rational nature of these variables in the cognitive process of health behavior decision-making.^49,50,52^ The level of risk people attribute to a certain health hazard and the perceived effectiveness of a behavior that is supposed to minimize this risk are likely to determine, at least to some extent, the chances of engaging in the suggested behavior. Our results supported this hypothesis. This finding shows that there are some “universal” factors that impact compliance regardless of social or cultural affiliation. As a result, policy-makers and health authorities should take these factors into account when communicating with the public, particularly during an on-going crisis, in which several health instructions have already been issued. Their statements should underscore the dangers of the disease and the effectiveness of the instructions in fighting the virus and limiting its spread.

 With regard to trust in government, our hypothesis was supported as well. We expected trust in government to be more strongly associated with compliance in the Arab minority group compared to the other groups. Indeed, we found that trust in government was mildly, yet significantly and positively associated with compliance in the Arab group, but had no association in the other social groups (Figure 1). This finding with regard to the Jewish social groups contradicts the existing literature.^39,46,105-107,115^ Respondents in these groups reported their intentions of complying with the presumably newly issued health instruction, regardless of their level of trust in the government that issued it.

 One explanation for this somewhat surprising finding is the particular characteristics of our test case. Israelis have faced many major, life-threatening challenges and crises since the establishment of the state of Israel and even prior to that point. A long series of wars and terror attacks, a result of the on-going Arab-Israeli conflict, have shaped the Israeli mentality and created a society with a strong sense of unity and solidarity, particularly in times of life-threatening crises.^116-118^ It is possible that in times like this, political and civic perceptions, including those about trust in the government, are put aside, or at least play a smaller part in shaping the average Israeli’s behavior. Nevertheless, in accordance with our hypothesis, the relationship between trust in government and compliance in the Arab minority was weak but apparent. One explanation, as mentioned above, is that the Arab population feels estranged from the Jewish majority group.^25,64,65^ Furthermore, the Arab minority’s national background and its identification with the Arab side of the Arab-Israeli conflict place it in an opposition, “outsider” position with respect to Israel’s society and government. In this type of relationship, trust in government remains a significant consideration in determining compliance with the behavior the government wants, even in times of crisis. However, not all Arabs in Israel take the position of being “outsiders.” Some want to integrate into Israeli society and its national institutions.^119^ This phenomenon may explain the higher levels of compliance intentions found in our study for Arabs who trusted the government compared to members of the Jewish groups with the same levels of trust in government (see Figure 1). When members of minority groups wish to integrate with the majority group, they are likely to have more institutional trust.^120,121^ They are also likely to conform with the institutions, norms and rules associated with the majority. Doing so may promote their integration by signaling to the majority group that they are trustworthy partners for social interaction.^122,123^ In the current case, it is plausible that Arab individuals who wish to integrate in the majority group of Israeli society have higher levels of trust in government. Thus, they might be more motivated to comply with government instructions than individuals from the majority group who have the same levels of trust in government because they want to make a good impression on the general society.

 As for the ability to comply, in accordance with our hypothesis this factor was differently associated with compliance intentions, depending on the social group being examined. As Figure 2 demonstrates, despites the weak effect size, this relationship was significant only with regard to the general population. The technical ability to follow the simulated instruction conditioned the intentions to comply with this instruction only in the general population, not in the other social groups. One explanation for this finding may be the experience of the Arab and ultra-Orthodox communities with previously issued health instructions that were incompatible with their lifestyles, but with which they generally did comply. In other words, these groups learned that directives that initially seemed impossible to comply with considering their lifestyle, could eventually be adapted in a way that made them easier to follow. Thus, perhaps in some cases, people get used to the mere notion of compliance, and not just the regulations themselves. This suggests a possibly more easily achieved compliance in more advanced stages of an on-going crisis. Some might argue that in these results we see the effect of social desirability bias. However, as mentioned above, since other well-established compliance-associated factors, such as perceptions about risk and effectiveness were associated with compliance in these social groups as well as in the general population, we contend that social desirability bias probably plays a limited role in our results. In addition, while it may seem counterintuitive that owning a thermometer is not related with compliance intentions, it is plausible that in this specific case, not owning a working thermometer may not be a significant barrier in enabling compliance. This situation could easily be changed by purchasing a new thermometer, which is affordable and accessible. In this regard, compliance intentions may latently include purchasing intentions.

 Interestingly, our study did not find a cross-group relationship between other known compliance-related demographic factors, such as age, gender, and income level and compliance intentions.^11,28,44^ However, other studies of compliance during COVID-19 also established that these factors were insignificant.^30^ Other commonly associated factors with compliance, such as stress, social capital, knowledge about the disease and satisfaction with the government’s performance during the pandemic were also not related with the compliance intentions in any of the samples. One explanation for this result is the unique and unprecedented circumstances of the current pandemic such as its global magnitude and high levels of uncertainty affecting all areas of life.^15,124^ These extreme conditions, may impact formerly established relationships between some variables and compliance.

 Finally, our findings highlight additional group-specific compliance related factors. In the Arab group, beyond trust in government, gender, education and income level were significantly related to compliance intentions. These findings emphasize the variance in the conditions associated with the behavioral intentions of different social group during a severe and prolonged health crisis. While in the Arab minority the factors that were associated with compliance intentions were the most similar to those in previous findings, the other groups demonstrated an association with compliance intentions for only the most basic compliance-enhancing factors. More importantly, our findings underscore that the association between trust in government and compliance intentions, which has repeatedly been reported in the literature, should be regarded as a factor that might be related to compliance only for specific social groups, at least when faced with an ongoing global crisis.

 Like any study, this study suffers from a number of limitations. First, our minority samples are not fully representative. Given the difficulty in accessing both the Arab and ultra-Orthodox minorities for research purposes,^18,91^ our samples include only basic demographic stratifications, not a full demographic representative profile. This is a common challenge in minority group research in Israel, and many studies report using samples that are only partially representative of the groups they are investigating.^65,72,125^ As a result, our findings should be treated with caution as they do not necessarily represent older minority group members who are less cooperative when approached to take part in research in general and online studies in particular. Second, it should be stressed that this study measured compliance intentions to a particular and a simulated newly issued health instruction. Therefore, it may be possible that a different instruction that may require other resources and efforts to comply with or entails sanctions of various extent for not complying with it, may yield different results. Third, the uniqueness of the social composition in Israel and its historical and political context challenge the external validity of our results and call for future multi-national research on compliance intentions with health measures. Furthermore, it should be stressed that our findings capture a specific point in time in the course of a prolonged pandemic. It is possible that other stages of this or other pandemics would yield different results. However, while the specific findings regarding each of the social groups could not be generalized to other groups in other countries, or to other specific health instructions in this or in different health crises, they do highlight the role of minority group affiliation in compliance with protective health measures during a pandemic. The variance in the compliance-enhancing factors between different minority groups stresses the importance of the unique features of social groups when developing and promoting preventive health measures. Different social groups should not be viewed as a uniform public, but rather as “apples and oranges” in the social fruit basket of a country. Hence, health instruction strategies and messages should be tailored to social groups based on their motivations and values. However, acquiring knowledge regarding the specific compliance-enhancing factors for each minority group in each crisis may be time consuming and impractical at the onset of the crisis. While these group-oriented adjustments may be practical and effective for more advanced stages of the crisis, in the initial stages, and particularly in ones that include the frequent issuing of new instructions, it may be more beneficial to emphasize the risks and dangers posed by the specific threat and the effectiveness of the promoted preventive measures. It is these factors that have proven to have a cross-social association.

## Conclusion

 This study suggests new insights regarding the factors related to compliance with newly issued health instructions during a pandemic. Our study indicates that, in times of crisis, while perceptions about risk and the effectiveness of proposed measures may have a similar association with compliance intentions in all social groups, other factors such as trust in government and the ability to comply with the instructions are differently associated with compliance intentions in other social groups. Despite the partially representative sample and our focus on a unique case study in a single stage of a pandemic, our findings question the frequent recommendation for policy-makers and health authorities to improve trust in government in order to achieve better compliance during a crisis.^126^ Such an approach may not be effective in extreme situations and for all social groups. A more effective information strategy would be underscoring the risks and dangers that the crisis may pose as well as the effectiveness of the recommended new behaviors for dealing with the crisis.

## Ethical issues

 The study was conducted following the approval of The Social Sciences faculty’s ethics committee of the University of Haifa, Israel (approval #112/20).

## Competing interests

 Authors declare that they have no competing interests.

## Authors’ contributions

 TG oversaw acquiring the data, drafting the manuscript and providing administrative and technical support. She also had a major contribution in obtaining the funding for this study, conceptualizing and designing this study, and analyzing the data. The second and third authors contributed equally. DRV took part in conceptualizing and designing the study’s procedure as well as analyzing and interpreting its data. She also contributed to obtaining the funding, supervising over the execution of the study and revising the manuscript. IB contributed to the study’s conceptualization and funding obtainment. He also supervised its performance and contributed to the interpretation of the data and the revision of the manuscript.

## Funding

 This research was supported by the Ministry of Science & Technology, Israel.

## Endnotes

 [1] The Israeli Ministry of Health’s official Telegram channel: https://t.me/MOHreport/4169.
[2] We checked for inter-sample differences in the demographic variables by testing for interaction effects of the social group on the relationships between the demographics and compliance intentions. A single significant interaction emerged when the Arab group was defined as the reference group: ultra-Orthodox X income level (b = 0.252; *P* < .05).

